# Life on Magnets: Stem Cell Networking on Micro-Magnet Arrays

**DOI:** 10.1371/journal.pone.0070416

**Published:** 2013-08-01

**Authors:** Vitalii Zablotskii, Alexandr Dejneka, Šárka Kubinová, Damien Le-Roy, Frédéric Dumas-Bouchiat, Dominique Givord, Nora M. Dempsey, Eva Syková

**Affiliations:** 1 Institute of Physics AS CR, v.v.i., Prague, Czech Republic; 2 Institute of Experimental Medicine AS CR, v.v.i., Prague, Czech Republic; 3 Institut Néel, CNRS/UJF, Grenoble, France; Rutgers - New Jersey Medical School, United States of America

## Abstract

Interactions between a micro-magnet array and living cells may guide the establishment of cell networks due to the cellular response to a magnetic field. To manipulate mesenchymal stem cells free of magnetic nanoparticles by a high magnetic field gradient, we used high quality micro-patterned NdFeB films around which the stray field’s value and direction drastically change across the cell body. Such micro-magnet arrays coated with parylene produce high magnetic field gradients that affect the cells in two main ways: i) causing cell migration and adherence to a covered magnetic surface and ii) elongating the cells in the directions parallel to the edges of the micro-magnet. To explain these effects, three putative mechanisms that incorporate both physical and biological factors influencing the cells are suggested. It is shown that the static high magnetic field gradient generated by the micro-magnet arrays are capable of assisting cell migration to those areas with the strongest magnetic field gradient, thereby allowing the build up of tunable interconnected stem cell networks, which is an elegant route for tissue engineering and regenerative medicine.

## Introduction

Our planet produces a small magnetic field, about 50 µT, which varies on a length scale much larger that the size of humans, animals and cells. Nevertheless, even a small and quite homogenous magnetic field is crucial for many aspects of the lives of both humans and microorganisms, e.g. left-right inversion in the human brain [1]; magnetoreception observed in magnetotactic bacteria and believed to occur in certain animals, such as birds. But what happens when a living cell interacts with a strong magnet of similar size to itself? The stray field produced by such a micro-magnet will dramatically change in value and direction across the cell body and the question is: how will the cell respond and adapt itself to a high magnetic field gradient? In spite of tremendous recent progress in cell biology and the ever growing use of magnetic materials in bio-medical applications, little is known of the long-term influence of a magnetic field at the cellular level. In studies of the effects of a magnetic field on living cells, mesenchymal stem cells are the subject of particular interest because of their ability to differentiate into adipocytes, chondrocytes and osteoblasts as well as other cell types [2], thus allowing tissue regeneration and providing therapeutic effects on diseases for which there is no other effective therapy. For tissue growth, the spatial organization of a stem cell colony and its geometrical and mechanical constrictions play an important role [3]–[5]. Thus, manipulating the fate of stem cells, their spatial organization and the creation of an interconnected cell network with externally applied magnetic fields is of great potential interest for tissue engineering applications. Here, we describe experiments with micro-magnets and living cells that reveal the dramatic impact of a high magnetic field gradient on the spatial organization and growth of stem cells. The observed magnetic control of the stem cells is discussed from the points of view of both physics and biology.

Let us start with a brief description of the relevant effects of a magnetic field on biological objects. The influence of a magnetic field on materials is a familiar process not expected to show surprises – an externally applied magnetic field can either pull or push an object depending on the sign of the object’s magnetic susceptibility (paramagnetic, ferromagnetic, ferrimagnetic and superparamagnetic objects being attracted, diamagnetic objects being repelled). In this sense, living objects – organisms, cells and biomolecules – are not different; nevertheless, due to their inherent complexity it is difficult to distinguish between the different types of magnetism inside a living cell. The forces and effects induced by magnetic fields may offer unique control of cell motion, proliferation and machinery as well as a new opportunity for promising applications ranging from micro/nano-scale control, such as cell sorting, drug and gene delivery [6], to controlling the behavior of animals [7] and even humans [1]. Depending on cell type, exposure to a low or moderate static magnetic field may either increase or decrease Ca^2+^ influx; for a review, see [8]. The possibility of monitoring and remotely controlling cellular endocytosis and/or exocytosis rates of superparamagnetic iron oxide (SPIO) nanoparticles using a magnetic field was recently demonstrated [9], [10]. A study of the direct influence of a magnetic field on a cell and the possibilities of magnetically controlling cellular motion, trapping and patterning, without the use of SPIO nanoparticles inserted in, or attached to the cells, is especially important because this approach avoids problems related to nanoparticle toxicity and removal. Such a *direct* influence of magnetic fields on living cells may exhibit itself in high magnetic field gradients, when the external magnetic field varies at the same scale as the cell size, i.e. in the close environment of micron-sized magnetic flux sources [11], [12]. Arrays of micro-magnets, which produce magnetic field gradients up to 10^6^ T/m [11], have indeed been used to diamagnetically trap arrays of Jurkat cells, in the presence of a paramagnetic contrast agent [13]. Such micro-magnets can also be used to attract and trap cells functionalized with SPIO nanoparticles [9], [14], [15].

In this work we studied the behavior of SPIO nanoparticle-free mesenchymal stem cells in standard medium without any added paramagnetic contrast agent, in the presence of high magnetic field gradients generated by patterned micro-magnets.

## Materials and Methods

### Micro-magnet Arrays

**Figure 1:**
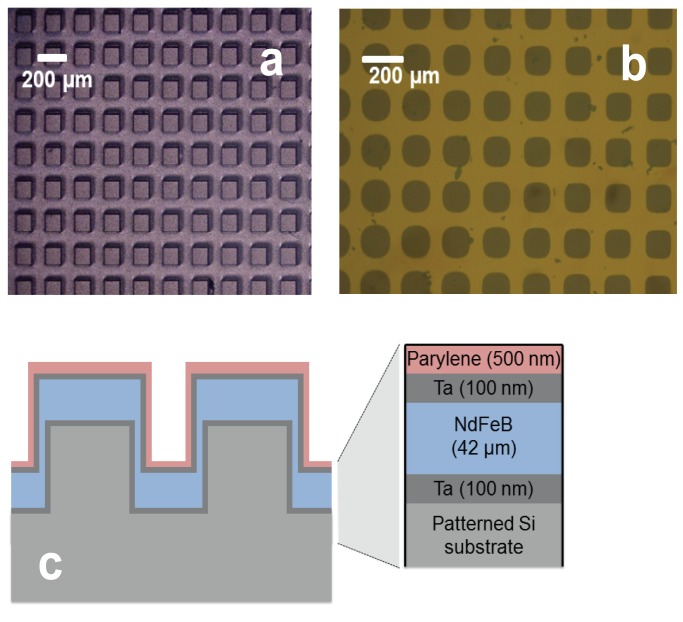
Square micro-magnets arrays. Plan-view (a) optical and (b) magneto-optical images of square micro-magnets (100×100 µm^2^) separated by 100 µm. In the latter case, a uniaxial Magneto-Optic Indicator Film placed upon the micro-magnet array serves to reveal the direction of the z-component of the stray field pattern produced above the micro-magnet array, following out-of-plane magnetization in a magnetic field. Side-view of micro-magnets (c).

Si substrates were patterned, using lithography and deep Reactive Ion Etching (RIE), to produce arrays of Si pillars of lateral size 100×100 µm^2^, 30×30 µm^2^ and 20×20 µm^2^ and depth of 100 µm, separated by a distance equal to the pillar width. High rate triode sputtering was used to deposit Ta (100 nm)/NdFeB (18 µm)/Ta (100 nm) trilayers at a temperature of 450°C, which were subsequently annealed at 750°C for 10 minutes. Such films display excellent hard magnetic properties (coercivity of the order of 1.5 T, out of plane remanent magnetization values of up to 1.2 T) [16]. As an example, optical and magneto-optical images of an array of 100 µm magnets are shown in Figure 1. The micro-magnet arrays were uniformly coated with a 500 nm layer of parylene, a biocompatible polymer film (Figure 1c). Ta is immiscible with both NdFeB and parylene at the temperatures concerned here [16], [17], ensuring no diffusion from either the NdFeB or Ta capping layer into the MCS culture medium. More information about the fabrication and characterization of the NdFeB micro-magnet arrays can be found in [11], [13], [16]. Arrays of bare Si pillars, used for control experiments, were also coated with a 500 nm layer of parylene.

### Cell Culture

Mesenchymal stem cells (MSCs) were isolated either from Wistar rats or from enhanced green fluorescent protein (EGFP) transgenic TgN (acro/act-EGFP)4Osb rats [18], which express EGFP under the control of a chicken promoter for β-actin and a cytomegalovirus enhancer. The cell isolation procedure from the animals was approved by the Central Commission for Animal Protection of the Academy of Sciences of the Czech Republic in Prague. The Institutional Animal Care and Use Committee specifically approved this study. Briefly, the bone marrow was extruded from the femurs using a needle and syringe with MSC culture medium containing Dulbecco's Modified Eagle Medium (PAA Laboratories, Pasching, Austria) with 10% fetal bovine serum (PAA Laboratories) and PrimocinTM (100 µg/ml, Lonza, Cologne, Germany). After 24 h in culture, the non-adherent cells were removed by replacing the medium. Cultures were kept in a humidified 5% CO_2_ atmosphere at 37°C; cells up to the third passage were used for seeding on magnet arrays. The magnet arrays were sterilized in 70% ethanol, washed in phosphate buffered saline (PBS) and placed into a 24-well plate with MSC culture medium. A suspension of 50,000 cells was seeded per array. After 4 hours, or 1–3 days, cells grown on the arrays were fixed in paraformaldehyde in PBS for 15 min and stained for actin filaments with Alexa-Fluor 568 phalloidin (1∶300, Invitrogen, Paisley, UK). The nuclei were visualized by using 4′,6-diamidino-2-phenylindole (DAPI, Invitrogen) fluorescent dye. The magnetic arrays were monitored on an AxioCam HRc Axioscop 2 Plus fluorescent microscope (Zeiss, Jena, Germany) and a LSM DUO5 laser scanning confocal microscope (Zeiss, Jena, Germany).

## Results

### Initial Adhesion of Cells (4 hours)

**Figure 2:**
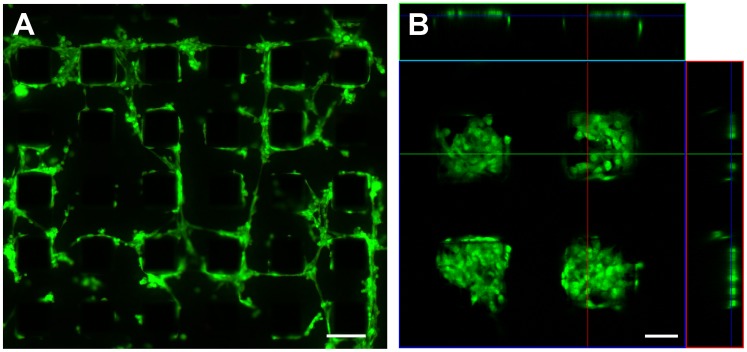
Cells on a micro-magnet array (100×100 µm) 4 h (A) and 3 days (B) after seeding. B: 3D-confocal views. The cells migrated to and proliferated on the tops of the micro-magnets. Scale bar: A: 100 µm, B: 50 µm.

**Figure 3:**
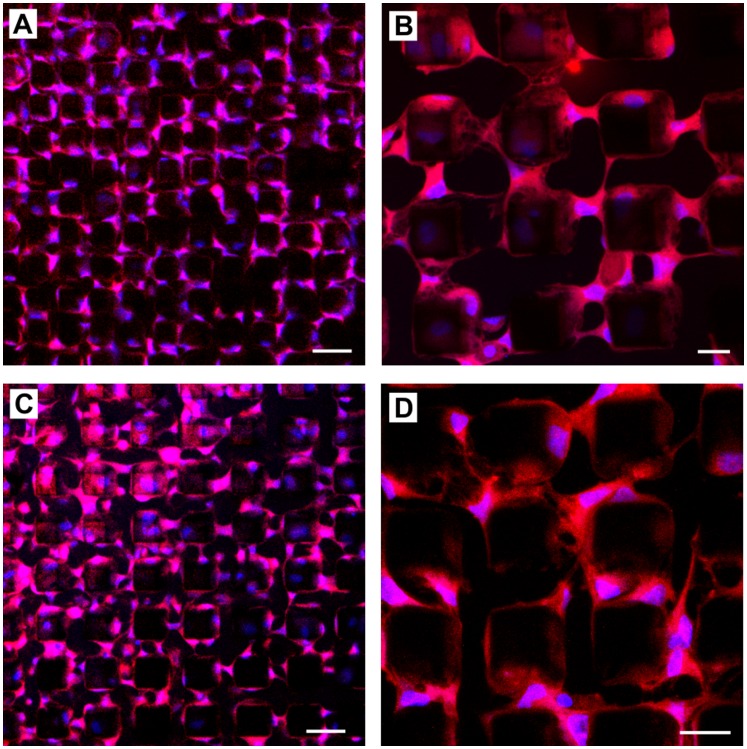
Confocal micrographs of the pattern of stem cells on micromagnets with periods of 20 µm (A, B) and 30 µm (C, D) 4 hours after seeding. The stem cells are stained for actin filaments (red) and cell nuclei (blue). The cells were mostly attracted to the walls and edges of the micro-magnets. Because the distance between the micro-magnets is almost the same as the cell size, the cells create bridges between the neighboring micro-magnets. Scale bar: A, C: 50 µm, B, D: 20 µm.

**Figure 4:**
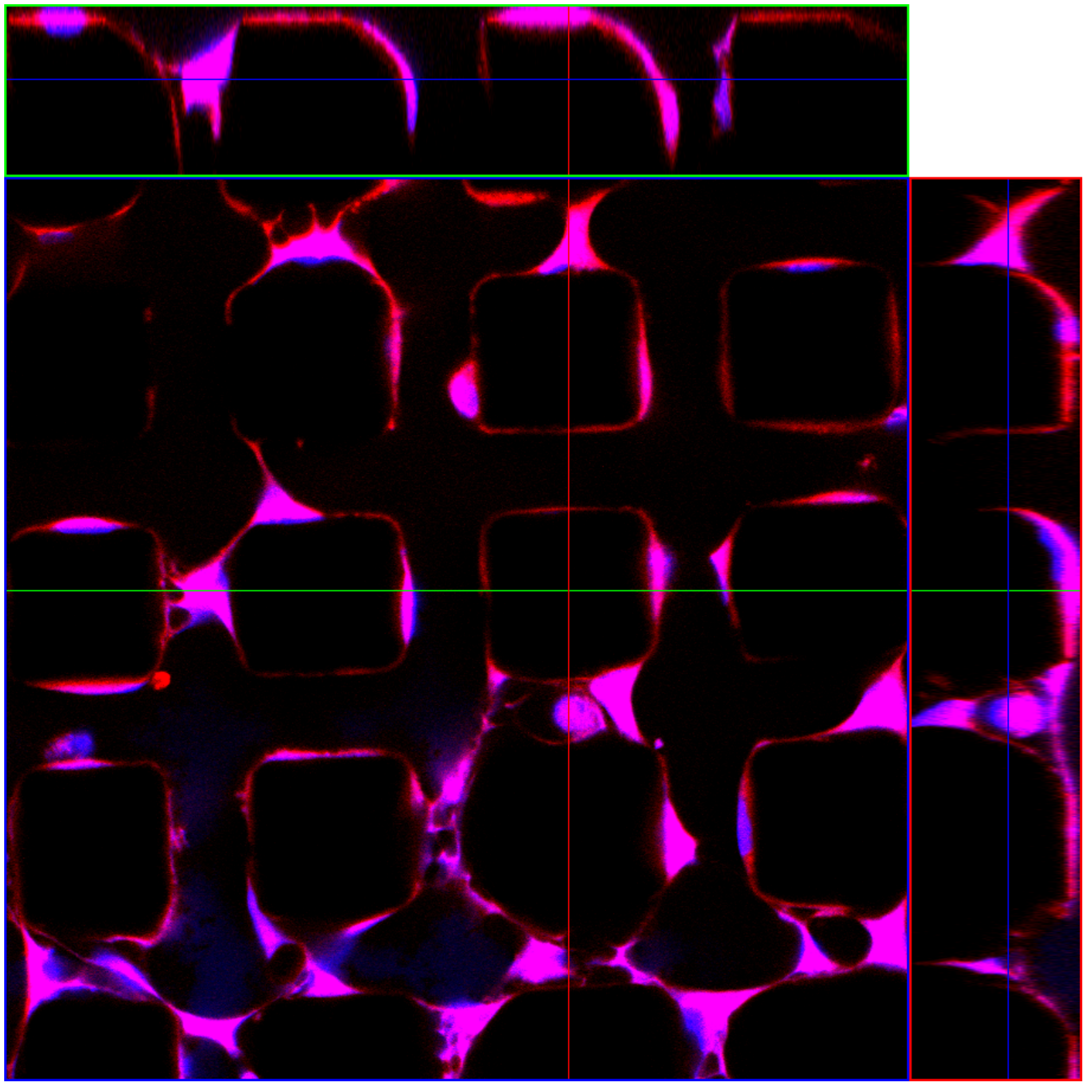
Confocal microscope image of stem cells on a micro-magnet array with a period of 30 µm 4 hours after seeding. The cells were stained for actin filaments (red) and cell nuclei (blue). A 3D-reconstruction of the shapes and positions of the cells is possible with the top and side views shown (see the images on the top and right side of the figure).

The stem cells were dropped onto micro-magnet arrays (Figure 1) with different feature sizes. Generally, cell adhesion on a substrate is provided by the binding of transmembrane integrin receptors to specific parts of molecules of the extracellular matrix, such as laminin, collagens or fibronectin. Factors affecting cell adhesion on a substrate include the chemical composition and charge of the surface, the surface roughness and topography, as well as the balance in surface ratio between hydrophilic and hydrophobic areas [18], [19]. To observe the initial response of cells to the magnetic field pattern produced by the micro-magnet array, cells were observed 4 hours after their deposition upon the array. This time interval is sufficient for cell adhesion on the micro-magnet array, during which cells change their spherical shape, which they assume in a suspension, to a spindle-like shape. It can be seen that cell positioning is not random. On the array of period 100 µm (Figure 2a), the cells were mostly attached to the edges of the micro-magnets while for the smaller period, 20 and 30 µm arrays (Figures 3, 4), the cells bridged neighboring micro-magnets (Figures 3b-d). The latter is a consequence of the fact that the cell size and the distance between neighboring micro-magnets are comparable. Two general features of cell adhesion on the micro-magnet arrays were observed: i) attraction of cells to the edges of the micro-magnets and ii) changes in their shape so as to be elongated parallel to the edges of the micro-magnets (Figures 2a, 3 and 4).

### Migration of Cells (1–3 days)

**Figure 5:**
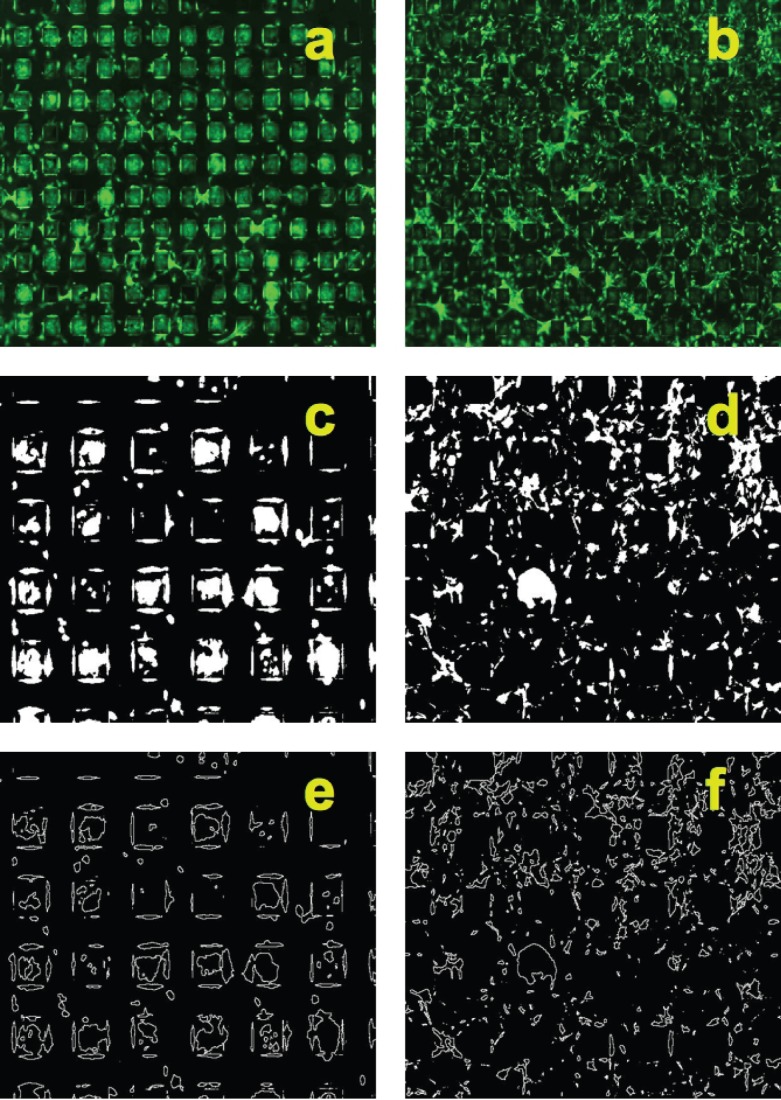
Stem cell networks on magnetic and non-magnetic arrays. Original fluorescent micrographs of cells cultured for 2 days on (*a*) magnetic and (*b*) non-magnetic arrays with a period of 100 µm (top panel). The stem cells are visualized by GFP protein. The cells grew and proliferated mostly on top of the micro-magnets. The cells on non-magnetic arrays grew preferentially between the Si pillars. Middle panel (c,d): binarized zoom of the top right corner of the original images (*a* and *b*). Bottom panel (e,f): the boundaries (perimeters) of the images (c) and (d) shown in the middle panel. Here, each object represents a cell cluster. The number of cell clusters equals 85 for magnetic and 433 for non-magnetic arrays, respectively.

After longer periods, e.g. 3 days (Figure 2b) or 2 days (Figure 5), the cells are positioned on the tops of the micro-magnets, creating patterns that reflect the geometry of the underlying micro-magnet arrays, with very few cells in the space between the micro-magnets. The final stage of the magnetically assisted stem cell migration – cells seated on the tops and edges of the micro-magnets - is shown by confocal microscopy images in Figures 4 and 5. This indicates that the cells preferred to migrate to and grow on the tops of micro-magnets, not in the valleys between them. The effects of substrate topography and micro-patterning on cell functions due to cell-substrate interactions were recently reported in [20]–[25]. In particular, human neural stem cells cultured on a silicon surface patterned with 25 µm deep micro-channels preferred to live inside the grooves of the micro-channels, not on top of them [26]. In order to distinguish between the influence of the surface topography and that of the magnetic field pattern of our micro-magnet arrays, we have cultured cells on two sets of arrays having the same surface topography (100 µm square features of depth 100 µm, separated by 100 µm), one “non-magnetic” (i.e. arrays of Si pillars), one “magnetic” (i.e. arrays of Si pillars with a NdFeB deposit on top). Both samples were covered with parylene. Figure 5 shows stem cells cultured on the non-magnetic and magnetic arrays for 2 days. As can be seen from Figure 5, ordered cell patterns appear only on the magnetic arrays, thus evidencing the magnetic nature of the observed effect. The images obtained after two steps of image processing (binarization and perimeter visualization) are shown in Figures 5b and 5c. As it can be seen from these figures, both clustering and ordering degrees of cell networks on magnetic and non-magnetic arrays are quite different. The number of cell clusters per image area is a convenient parameter to characterize the magnetic influence on cell networking. The estimation of the number of cell clusters (that are formed by treating as connected only non – corner neighboring pixels) gives 85 and 433 for magnetic and non-magnetic arrays, shown in Figure 5 (bottom panel), accordingly. Thus, the magnetic array influence on the cell networking manifests itself in the three distinctive features of networks: i) creation of big enough cell clusters, ii) inheritance of four-fold symmetry of the magnetic array and iii) cell positioning on the top of micro-magnets.

To understand the physical and/or biological (biochemical) reasons for the guided migration of stem cells on micro-magnet arrays, we considered the forces that may act on the cells in a medium. Estimations of the Lorentz and magnetic gradient forces may give a first hint as to which force might be responsible for the cell patterns observed.

## Discussion

The primary aim of this study was to investigate whether the highly inhomogeneous magnetic field patterns produced by micro-magnet arrays, could promote the alignment of stem cells and, if so, to reveal the underlining mechanisms. To the best of our knowledge, this is the first report on the effects related to the direct influence of a magnetic field on cellular life. Below, by analyzing a few hypothetical mechanisms that might be responsible for the observed effects, we show that cell patterning is the result of the interplay of physical and biological factors.

### Magnetic Gradient Force Attracts Cells to the Edges of Micro-magnets

The magnetic gradient force density (in N/m^3^) acting on an individual cell is
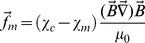
(1)where χ_m_ is the susceptibility of the medium and χ_c_ is the susceptibility (paramagnetic or diamagnetic) of the cell, μ_0_ is the vacuum permeability, and *B* is the magnetic induction. Cells, and typical cell media, are generally considered to be diamagnetic. However, due to a difference between the intracellular and extracellular concentrations of paramagnetic ions, a cell may be less diamagnetic than the surrounding medium. Such a difference in the susceptibilities we denote as Δχ = χ_c_-χ_m_. Thus, for Δχ>0, the cells are attracted to the areas with the highest magnetic field gradient, while for Δχ<0, the cells are repelled from them. Having no precise data on the susceptibility of the given cells, we turn to the experiment shown in Figure 3. Here the cells create bridges between two neighboring micro-magnets. As seen from Figures 3 and 2a, the cells also prefer to adhere to the top edges of micro-magnets. Moreover, observations show that the cells initially adhere to the edges of the micro-magnets, and then migrate to the top of the micro-magnets. These observations imply that these cells are attracted to the areas with the highest magnetic field gradient - the edges of the micro-magnets, i.e. Δχ>0 in Equation 1.

**Figure 6:**
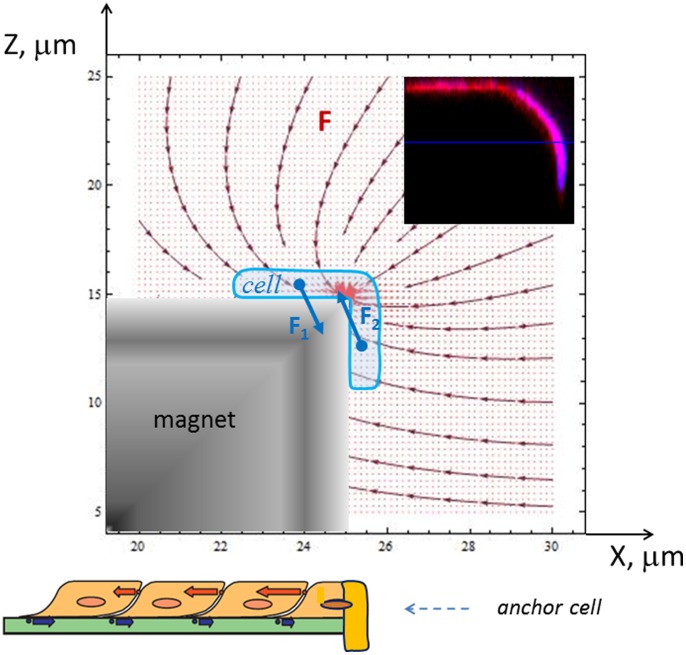
Magnetic field, forces and cell position on a micro-magnet. Magnetic force distribution and force directions acting on a cell adhered to the edge of a micro-magnet calculated for a magnet of size: 50×50×30 µm. Side view shows the area near the edge of the micro-magnets. The plotted forces are ***f_m_*** = (Δχ/μ_0_)(**B∇**)**B** normalized to Δχμ_0_M_S_^2^/*a*, where *M_S_* is the saturation magnetization of the magnet and *a* is the lateral half-size of the magnet. The inset of the confocal microscope image shows the shape and position of a cell (see Figure 4). The bottom panel shows a sketch of the tug-of-war model with an anchor cell and the direction of cell proliferation. The red arrows represent the traction forces. The inset shows a confocal image of the cell’s position at the micro-magnet edge.

Keeping in mind that the forces are attractive, let us consider the balance of forces acting on a cell adhered to the edge of a micro-magnet. We now discuss how the cell colonies (shown in Figures 2–5) are formed on top of a micro-magnet. In this process, two steps can be distinguished. Firstly, a primary cell attracted to a micro-magnet edge should stay on the micro-magnet, as shown in Figure 6. Close to the edge, the cell adopts the shape caused by the tug exerted by both magnetic planes: XY and YZ. The corresponding resultant magnetic forces, *F_1_* and *F_2_*, exerted on both parts of the cell are shown in Figure 6. The magnetic force balance can only be satisfied for such a bent cell shape and position. Secondly, the living cells adhered to the edge start to proliferate and newly appearing cells migrate to the center of the top surface of the micro-magnet, as observed. The forces involved in this migration are the magnetic gradient force, tether (adhesion to the substrate) and the traction forces generated by the cell. One can neglect the gravitational force which is balanced by the buoyancy force. The crucial question concerning the conditions and the directions of the colony’s growth is: may the magnetic force either assist or oppose the cell traction forces? A comparison of the traction, magnetic and adhesion forces, presented in the Appendix S1, leads us to conclude that magnetically assisted cell migration is possible. In [27] the growth of a cell colony is described in terms of an advancing cell sheet characterized by traction force distributions and the averaged surface cell traction stress. Thus, we calculate the averaged cell stress caused by the magnetic force as *σ_m_ = f_m_L*, where *L* is the cell length.

So, assuming that *L* = 20 µm and estimating Δχ to be 10–20% (see the Appendix S1) of the diamagnetic susceptibility of water (−9 ⋅10^−6^ in SI), B = 1 T and ∇B = 10^6^ T/m, from Eq. 1 we get the magnetic force exerted on a whole cell, *F_m_ = f_m_L^3^* = (6–12) nN, and then from σ_m_ = *f_m_L* one can obtain σ_m_ = (10–30) Pa. This value is comparable to the traction stress ranging between 50–300 Pa (depending on the distance from the leading edge) within the cell sheet reported in [27] for kidney epithelial cells. Thus, the cell migration and the direction of cell proliferation can be affected by the magnetic force gradient. In other words, in our experiments the cell culture sheet can be influenced by either compressive or tensile magnetic stress (stress within the cell culture sheet on a plane perpendicular to the substrate and parallel to the leading edge). The estimated magnetic stress σ_m_ = (10–30) Pa is also comparable to (or larger than) the flow-induced cytoskeletal stress (σ_cyt_) in living cells, which was found to be of the order of 1Pa in [28], and to the internal cytoskeleton stress (0–400 Pa) modulated by a pre-stress reported in [29]. So, when σ_m_>σ_cyt_ for the cells sitting on the micro-magnet edge (e.g. shown in Figure 5), the compressive magnetic stress can lead to cell elongation in the OY-direction (along the micro-magnet edge; see Figure 6) because of a large value of the Poisson ratio, ν (e.g. ν = 0.42 measured for THP-1 cells [30]). In turn, the elongated cell shape determines the direction of cell proliferation and spreading: perpendicular to the magnet edge towards the magnet center.

### Does the Lorentz Force Influence Membrane Channels?

**Figure 7:**
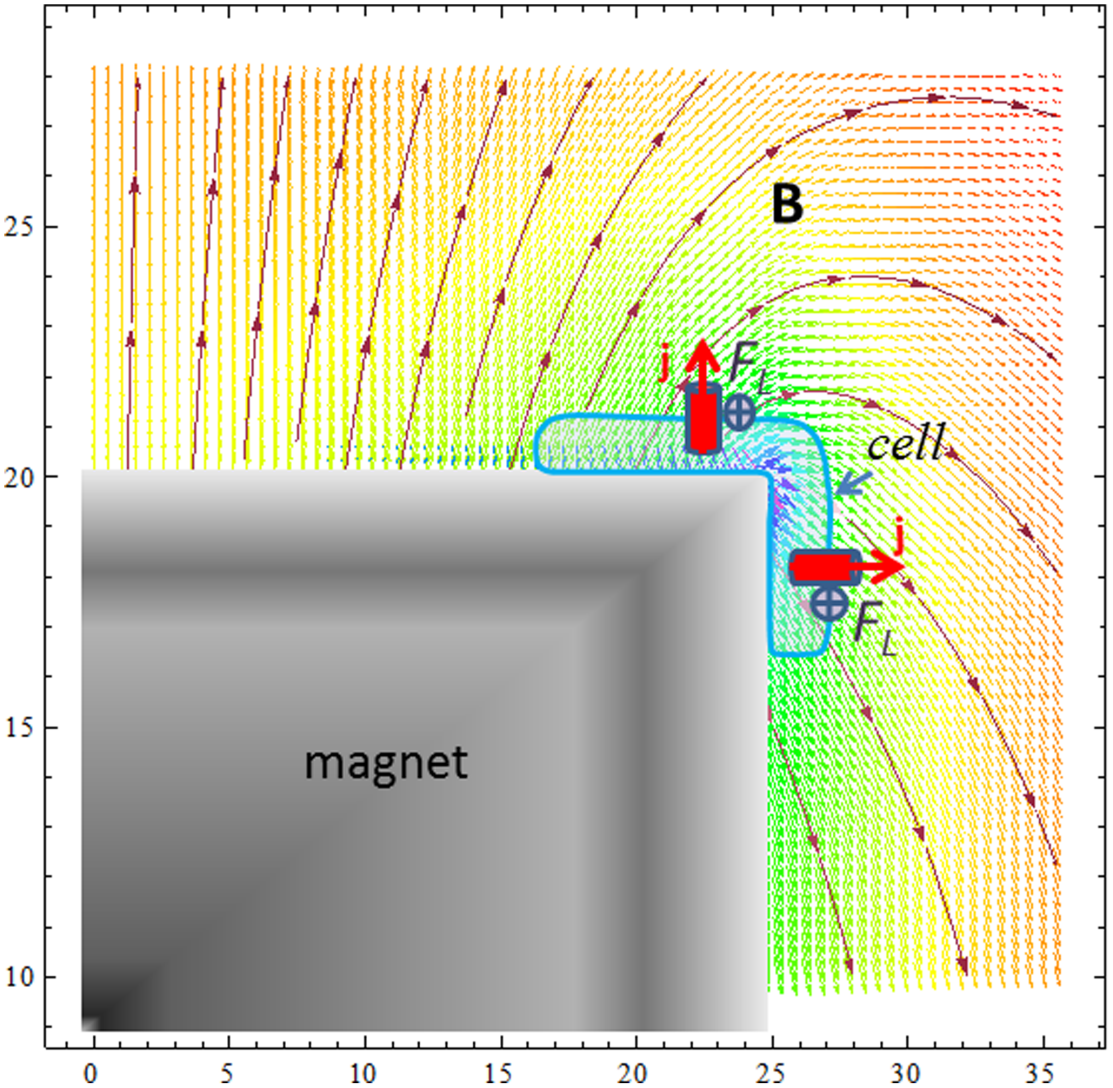
Magnetic lines near a micro-magnet calculated for a magnet of size 50×50×40 µm. Side view shows a quarter of the micro-magnet. All of the lengths are in micrometers. Two ion channels are shown in red. The Lorentz forces shown are perpendicular to the figure plane.

One of the possible mechanisms underlying many magneto-biological effects is the opening or closing of mechanically sensitive membrane ion channels. These channels operate at the thermal fluctuation energy limit, *k_B_T*, and therefore a small external input of mechanical energy provided by, e.g., magnetically induced membrane stress, will change the probability of a channel opening or closing. In ion channels the Lorentz force density can be estimated knowing the current density, which is j = I/πr^2^ = 10^8^ A/m^2^ (for a typical current *I* = 10 pA in a channel of radius *r* = 0.1 nm). Thus, in an ion channel the Lorentz force volume density for *B* = 1 T is *f_L_ = jB* = 10^8^ N/m^3^. One can compare these forces with the gravitational force density, *f_g_ = ρg* = 10^4^ N/m^3^, which yields the following: *f_L_>>f_g_*. An additional membrane local *stress* arising due to the Lorentz force is σ_L_ = *jBπr^2^d/2rd = IB/2r,* where *d* is the membrane thickness. From the last formula estimations made for a typical ion current *I* = 1.6 pA and radius *r* = 0.1 nm gives *σ_L_* = 0.8⋅10^−2^ Pa for *B* = 1 T. This stress multiplied by the membrane thickness, *σ_L_d* = 0.8⋅10^−2^⋅5⋅10^−9^ = 4⋅10^−11^ N/m, can be compared with the membrane *tension* known from literature: (10^−3^–10^−7^) N/m [31]–[33]; there is a difference of 5 orders of magnitude, at least. However, the membrane tension (*σ_L_d*) caused by the Lorentz force should be multiplied by the number of simultaneously active ion channels, *N*. This value is unknown, but its maximum is of the order of *N_max_ = (R/r)^2^* = 10^8^ (where *R* and *r* are the cell and ion channel radii, respectively). Moreover, the ion channel current can be up to 1000 pA (not 10 pA as assumed for the above estimations), and this also increases the possible value of the Lorentz stress on the membrane. Therefore, the question whether there is an effect of the Lorentz force remains open. In summary, if the membrane tension (σ_L_ *d*) caused by the Lorentz force is of the same order of magnitude as the cell membrane tension, for a cell sitting on the micro-magnet edge (as shown in Figure 6 and Figure 7) the Lorentz force may lead to a deformation of the cell shape along the magnet edge. Since all cell functions that involve membrane deformation or a change in cell shape (e.g., endocytosis, exocytosis, cell motility, and cytokinesis) are regulated by membrane tension, the role of an additional membrane tension caused by the Lorenz force might be crucial for cell life and proliferation.

### Paramagnetic Ions near Micro-magnets and their Circular Micro-flow

Close to the magnet surface one can expect an effect of the Lorentz force on magnetic fluid (a medium with paramagnetic ions) micro-motion, if the ratio between the Lorentz and magnetic gradient forces is about unity and the concentration gradient of the paramagnetic ions is large enough [34]–[36]. Indeed, close to a magnet surface where an ion concentration gradient exists, the Lorentz force is able to induce local convective flows [35]. These may exhibit themselves as a circular ion flow, e.g., a flow along the micro-magnet perimeter. Indeed, a primary ion flow towards a micro-magnet top causes a small magnetic gradient force, due to the Lorentz force becoming azimuthal. Such a secondary circular flow may assist the observed cell alignment along the magnet edges as well as their elongated shape, see Figure 2. Moreover, near the magnet edges an increased ion concentration arises because here the magnetic field gradient (and therefore the magnetic force) reaches its maximal value. For the cells, an increased ion concentration may serve as an additional stimulus for the observed cell alignment. In the observed cell behavior, the role of the Lorentz force is a subject of debate, mainly because of uncertainty about the value of the ion current and the number of active ion channels in the studied cells. Nevertheless, in a medium, near the top of the magnet both the Lorentz and the magnetic gradient forces can redistribute paramagnetic ions and/or chemoattractants and therefore create a pattern of chemical attractants needed for cell viability and proliferation.

### Conclusions

A tentative explanation of the observed cell alignment is the following: in the presence of a high magnetic field gradient, the cells’ behavior appears to be paramagnetic because, probably, the cells are less diamagnetic than the surrounding medium. Due to the magnetic forces the cells are attracted to the areas with the highest magnetic field gradient – the magnet’s edges – and adhere there. The magnetic stress causes the cells to elongate in the direction parallel to the magnet edge. The cells located on the magnet edge are bent across the edge in order to reach equilibrium with respect to the magnetic forces. The most strongly adhered cells constitute squares located along the magnet perimeters and serve as anchors for subsequent cell proliferation towards the magnet center. However, the cells are not independent identities; they intercommunicate in order to create a network for survival and spreading. The initial interconnected square network of cells is shown in Figure 2a, and this represents the starting structure for the colony to spread towards the magnet center. Indeed, keeping in mind that chemical communication between cells is established by special molecular cues, it is obvious that due to the geometrical constrictions, the cues’ concentration within the square is higher than that outside the square. This difference in concentration may be considered as the main reason for the observed cell proliferation towards the magnet center. Of course, during a cell’s life, the stress caused by the magnetic forces can affect all cell functions that involve membrane deformation or a change in cell shape (e.g., endocytosis, exocytosis, cell motility, and cytokinesis) and such effects are the subject of a forthcoming study.

The discovered cell patterning has a great application potential. Manipulating the fate and spatial organization of stem cells and the creation of an interconnected cell network with externally applied magnetic fields opens exciting perspectives for tissue engineering and regenerative medicine. For example, since cell seeding is the main step in engendering tissue structures in 3D scaffolds, micro-magnet arrays may be used as building blocks for new types of magnetic scaffolds, allowing us to enhance the adhesion, proliferation, and function of magnetic nanoparticle-free cells. Moreover, the magnetic stress arising near micro-magnets may serve as a tool driving stem cell differentiation, in a similar way as cyclic mechanical stress of 10–20 Pa applied to embryonic stem cells induces their differentiation [37].

Finally, the magnetic patterning of magnetic nanoparticle-free cells offers many opportunities for further theoretical and experimental research in biophysics, where the quantitative relationships between the imposed magnetic fields and the intracellular processes underlying cell life remain elusive.

## Supporting Information

Appendix S1 Magnetically Assisted Cell Migration and Estimation of the Difference between the Magnetic Susceptibilities. (DOC)
