# Supplementary material for: Life on Magnets: Stem Cell Networking on Micro-Magnet Arrays
Source: PLoS One. 2013 Aug 1;8(8):e70416. doi: 10.1371/journal.pone.0070416 (PMC3731273; doi:10.1371/journal.pone.0070416)
Supplement: Appendix S1 — (DOC) [file pone.0070416.s001.doc]

**Appendix S1**

*Magnetically Assisted Cell Migration*

Here we outline the role of magnetic forces in cell spreading. The process of cell spreading can be divided into three stages: 1) cell leading edge growth; 2) adhesion (tether) to the substrate and 3) cell body translocation. Note that the bent cells adhered to the magnet edge serve as an anchor for the spreading row of cells (see the bottom panel in Figure 6). If the planar magnetic force component exerted on a cell (directed towards the magnet edge) is less than the tether *F_tet_* (adhesion) force, one cannot expect an influence of the magnetic force when the second stage of cell spreading is completed. However, the tether force is time-dependent through the number of adhesion sites, which varies during cell movement. So, even if *F_m_<F_tet_* during the first and second stages, the magnetic force can assist or oppose the cell’s movement. A comparison of the above estimated magnetic forces (6-12 nN) and tether forces taken from the literature (e.g. for NG13 cells the tether force varies from 0 up to 0.4 nN [1]) implies a possibility *F_m_>F_tet_*. Of course, in the last inequality the sign depends on the substrate properties, cell type, magnetic gradient etc. Thus, the magnetic force may assist or oppose the third stage of cell spreading - cell body translocation. Indeed, at this stage the cell body is moved by relatively small forces due to cytoskeletal reorganization.

*Estimation of the Difference between the Magnetic Susceptibilities*

Below we estimate the difference in volumetric magnetic susceptibilities of a living cell and water, (χ_c_ -χ_w_). It is known that about 99% of cellular molecules are water molecules, with water normally accounting for approximately 70% of the total wet-weight of the cell. Since, by definition, the magnetic susceptibility is χ_c_=∑***p****_i_*/(*H* *V*) (where *H* is the magnetic field strength, ***p****_i_* is the magnetic moment of a cellular chemical substance, *V* is the cell volume and the sum is taken over magnetic moments of all chemical substances of a cell), the susceptibility of a cell can be divided into two parts: χ_c_=χ_w_+∑***p****_i_*/(*H V*), where the sum is taken over the volume occupied by paramagnetic and diamagnetic cellular chemical substances except water. Estimating the difference, (χ_c_-χ_w_) with the last expression, one should take into account the two following facts: i) the contributions of the remaining diamagnetic and paramagnetic cellular substances can fully or partially compensate each other and ii) the remaining volume comprises only 1% of the total cell volume. Thus, (χ_c_-χ_w_)/χ_w_ <<1 and this is a consequence of the high water concentration in a cell. A single paramagnetic molecule can cancel the diamagnetism of 100-1000 of water molecules. However, the concentrations of paramagnetic species are too small to overcome the dominant diamagnetism of a cell or tissue [2].  The order of the ratio, (χ_c_-χ_w_)/χ_w_ depends on the actual type of cells and medium. Unfortunately, very scarce literature data are available on magnetic susceptibilities of living cells. In [3] the difference between the magnetic susceptibilities of HeLa tumor cells and the medium was found to be Δχ/χ_w_=0.1. This ratio of the same order of magnitude was also found for Jurkat lymphocyte cells with the minimal concentration of paramagnetic contrast agent added in the cell medium [4]. It is important to note that some proteins have a similar magnetic susceptibility difference relative to water, about 10% [5,6] and lipids are diamagnetic with the susceptibility near that of water [2]. Thus, since water is the predominant component of most biological tissues, the magnetic susceptibility of most tissues appears to be 10-20 % that of water [2]. In light of the above, in Eq.1 we assume Δχ to be (10-20) % of the water susceptibility.

**References**

1. Nambiar R, McConnell RE, Tyska MJ (2009) Control of cell membrane tension by myosin-I. Proc Natl Acad Sci U S A. 106: 11972-11977.

2. Schenck JF (1996) The role of magnetic susceptibility in magnetic resonance imaging: MRI magnetic compatibility of the first and second kinds. Med. Phys. 23(6): 815-850.

3. Kashevskii BE, Kashevskii SB, Prokhorov IV, Aleksandrova EN, Istomin YP (2006) Magnetophoresis and the magnetic susceptibility of HeLa tumor cells. Biophysics 51(6): 902-907.

4. Kauffmann P, Ith A, O'Brien D, Gaude V, Boué F, et al. (2011) Diamagnetically trapped arrays of living cells above micromagnets. Lab Chip 11: 3153-3161.

5. Okada H, Hirota N, Matsumoto S, Wada H (2012) A simulation study of magnetic force effects on solution flow during protein crystal growth. J. Appl. Phys. 111: 093907.

6. Luo J, He X, d'Avignon DA, Ackerman JJH, Yablonskiy DA (2010) Protein-induced water 1H MR frequency shifts: contributions from magnetic susceptibility and exchange. J Magn. Reson. 202(1): 102.
